# Temporal and brain region‐specific elevations of soluble Amyloid‐β_40‐42_ in the Ts65Dn mouse model of Down syndrome and Alzheimer’s disease

**DOI:** 10.1111/acel.13590

**Published:** 2022-03-15

**Authors:** Savannah Tallino, Wendy Winslow, Samantha K. Bartholomew, Ramon Velazquez

**Affiliations:** ^1^ Arizona State University‐Banner Neurodegenerative Disease Research Center at the Biodesign Institute Arizona State University Tempe Arizona USA; ^2^ School of Life Sciences Arizona State University Tempe Arizona USA; ^3^ Arizona Alzheimer’s Consortium Phoenix Arizona USA

**Keywords:** Amyloid‐β_40‐42_, basal forebrain, down syndrome, hippocampus, Ts65Dn

## Abstract

Down syndrome (DS) is a leading cause of intellectual disability that also results in hallmark Alzheimer's disease (AD) pathologies such as amyloid beta (Aβ) plaques and hyperphosphorylated tau. The Ts65Dn mouse model is commonly used to study DS, as trisomic Ts65Dn mice carry 2/3 of the triplicated gene homologues as occur in human DS. The Ts65Dn strain also allows investigation of mechanisms common to DS and AD pathology, with many of these triplicated genes implicated in AD; for example, trisomic Ts65Dn mice overproduce amyloid precursor protein (APP), which is then processed into soluble Aβ_40‐42_ fragments. Notably, Ts65Dn mice show alterations to the basal forebrain, which parallels the loss of function in this region observed in DS and AD patients early on in disease progression. However, a complete picture of soluble Aβ_40‐42_ accumulation in a region‐, age‐, and sex‐specific manner has not yet been characterized in the Ts65Dn model. Here, we show that trisomic mice accumulate soluble Aβ_40‐42_ in the basal forebrain, frontal cortex, hippocampus, and cerebellum in an age‐specific manner, with elevation in the frontal cortex and hippocampus as early as 4 months of age. Furthermore, we detected sex differences in accumulation of Aβ_40‐42_ within the basal forebrain, with females having significantly higher Aβ_40‐42_ at 7–8 months of age. Lastly, we show that APP expression in the basal forebrain and hippocampus inversely correlates with Aβ_40‐42_ levels. This spatial and temporal characterization of soluble Aβ_40‐42_ in the Ts65Dn model allows for further exploration of the role soluble Aβ plays in the progression of other AD‐like pathologies in these key brain regions.

## Graphical abstract

This work characterizes the levels of soluble amyloid β_40‐42_ in the commonly used (Jackson Laboratory Strain #005252) Ts65Dn mouse model of Down syndrome across multiple brain regions and ages. Trisomic mice accumulate Aβ_40‐42_ in the basal forebrain, frontal cortex, hippocampus, and cerebellum in an age‐specific manner, with elevation in the frontal cortex and hippocampus as early as four months of age. Female trisomic mice also accumulate significantly more Aβ_40‐42_ within the basal forebrain than males by 7–8 months. The levels of the amyloid precursor protein in the basal forebrain and hippocampus inversely correlate with soluble Aβ_40‐42_ levels.

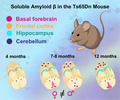

## 1 INTRODUCTION

Down syndrome (DS), which currently occurs in one out of every 700 births, is the most common cause leading to intellectual disability, resulting from a triplication of HSA21. By 40 years of age, nearly all DS patients develop the hallmark neuropathologies of Alzheimer's disease (AD), including extracellular amyloid beta (Aβ) plaques and intracellular neurofibrillary tangles (NFTs) of hyperphosphorylated tau (Head et al., 2012; Snyder et al., 2020). Notably, some estimates suggest that 90% of DS patients will go on to develop dementia by age 70 (Martinez et al., 2021). AD pathology in DS may be due in part to the increased production of the amyloid precursor protein (APP) and other proteins implicated in AD that lie within the region of HSA21 known as the DS critical region (DSCR). The formation of amyloidogenic Aβ plaques is a direct result of sequential cleavage of APP by β‐ and then γ‐secretases producing Aβ_40‐42_ monomeric fragments, which then oligomerize and further aggregate into insoluble plaques (reviewed in Masters et al., 2015). More recently, the accumulation of soluble Aβ fragments has been implicated as a driver of impaired synaptic activity and neurotoxicity in AD (Li & Selkoe, 2020).

Ts65Dn mice (Davisson et al., 1993) are one of the most widely used mouse models of DS as they include 2/3 of the gene homologues of the DSCR in the murine chromosome 16 (Ahmed et al., 2012; Antonarakis et al., 2004). These mice develop increased APP expression and protein levels, hyperphosphorylation of tau, and altered adult neurogenesis (reviewed in Hamlett et al., 2016); this mouse model also shows deficits in hippocampus‐dependent learning and memory (Costa et al., 2010). However, Ts65Dn mice do not develop insoluble Aβ plaques or NFTs as is typical of AD mouse models that carry humanized transgenes mimicking familial AD—this makes the Ts65Dn model an attractive tool to understand mechanisms contributing to neuropathology resulting from DSCR triplication, including the formation of various soluble lengths of Aβ. Past studies have explored APP levels and corresponding levels of soluble Aβ in Ts65Dn mice, but almost exclusively within the hippocampus and cortex (Ahmed et al., 2012; Choi et al., 2009; Hunter et al., 2003, 2003, 2004; Netzer et al., 2010; Sansevero et al., 2016; Seo & Isacson, 2005). Thus, a complete picture of region‐specific changes in soluble Aβ_40‐42_ as a function of aging has yet to be determined.

Trisomic Ts65Dn mice show elevated APP levels versus their euploid counterparts in the cortex, hippocampus, and cerebellum between 4.5 and 8 months of age (Ahmed et al., 2012). Additionally, trisomic mice show an age‐dependent increase of APP in the cortex, striatum (Hunter et al., 2003), and hippocampus (Hunter et al., 2003; Seo & Isacson, 2005). Male trisomic mice show increased soluble Aβ_40‐42_ in the hippocampus by 6 months of age as compared to euploid littermates (Hunter et al., 2003, 2004). It has also been shown that APP mRNA and protein levels increase in whole brains as a function of age, with greatest increases in the hippocampus and temporal cortex at 12 months; trisomic mice also show elevated soluble APP species (i.e., those cleaved by both α‐ and β‐secretases) at 12 months of age, but show no differences from their euploid littermates when looking specifically at soluble Aβ_40‐42_ from full‐hemisphere homogenates (Choi et al., 2009). Notably, the above studies were completed in the previous strain of Ts65Dn (Jackson Laboratory Strain #001924) with the recessive *Pde6b^rd1^* mutation, which leads to retinal degeneration and blindness in a subset of pups. The updated Ts65Dn strain (Jackson Laboratory Strain #005252) was bred to be homozygous for the wild‐type *Pde6b* allele and thus avoid the complication of retinal degeneration, and while the behavioral performance for the updated strain has been validated to be essentially unchanged from the original (Costa et al., 2010), the updated strain has also been reported to diverge anatomically and phenotypically from the original strain (Shaw et al., 2020). Studies of APP pathology in the updated strain so far demonstrate elevated soluble Aβ_40‐42_ in hippocampal homogenates of female trisomic mice at 12 months of age (Sansevero et al., 2016), and increased APP as well as c‐terminal fragments (resulting from β‐secretase processing just prior to γ‐secretase cleavage) and increased soluble Aβ_40‐42_ in trisomic hemibrain homogenates in females at 4 months (Netzer et al., 2010).

Importantly, a complete picture of age‐specific soluble Aβ_40‐42_ accumulation, including any sex‐specific differences, remains to be examined in the Ts65Dn 5252 strain. Additionally, various brain regions which show distinct abnormalities at different ages in Ts65Dn mice remain to be examined in relation to soluble Aβ_40‐42_ accumulation. For example, trisomic mice develop adult‐onset degeneration of basal forebrain cholinergic neurons (BFCNs) as early as 6 months of age (Granholm et al., 2000; Millan Sanchez et al., 2012), which recapitulates the very early alterations in this region seen in both AD and DS (Martinez et al., 2021). The triplication of APP and other surrounding genes in murine chromosome 16 is necessary to induce the early endosomal abnormalities seen at the start of BFCN degeneration in Ts65Dn mice (Cataldo et al., 2003; Millan Sanchez et al., 2012). It is thought that the more direct toxicity of soluble Aβ_40‐42_ may be particularly relevant in the degradation of sensitive BFCN populations (Nyakas et al., 2011). Also, while some disagreement remains as to whether or not the frontal lobe in DS brains shows hypocellularity (Pinter et al., 2001), patients with DS show pronounced executive functioning deficits (Tungate & Conners, 2021) and the Ts65Dn mouse model shows impaired prefrontal‐hippocampal circuit function around 3 months of age (Alemany‐González et al., 2020), which is mediated by input from the basal forebrain. Lastly, disrupted cerebellar development is also a common attribute in DS individuals, and Ts65Dn mice also show disrupted development of the cerebellar granule cell layer (reviewed by (Moldrich et al., 2007)). Intra‐cellular total Aβ accumulation in cerebellar Purkinje cells of Ts65Dn mice leads to axonal degeneration at 10 (Necchi et al., 2008) and 12 (Lomoio et al., 2009) months of age, possibly related to observed deficits in proteasomal clearance (Necchi et al., 2011), but it has not been shown which Aβ species contributes to these observations.

The levels of Aβ_40‐42_ in the basal forebrain and cerebellum have not yet been quantified within the Ts65Dn mouse model (1924 or 5252 strains) at any time point, and non‐specific cortical homogenates used in previous studies cannot reveal the changes in soluble Aβ_40‐42_ that may occur specifically in the frontal cortex, where attention and executive function are mediated via basal forebrain cholinergic input. The goal of this work is to characterize soluble Aβ_40‐42_ in multiple disease‐relevant brain regions of the updated Ts65Dn strain as a function of age, focusing on the basal forebrain, frontal cortex, hippocampus, and cerebellum. Additionally, we examine whether sex differences exist across the temporal and spatial spectrum in relation to soluble Aβ_40‐42_. This work will inform scientists on the sex‐, age‐, and brain region specificity of soluble Aβ_40‐42_ as future treatment‐based experiments are explored.

## 2 RESULTS

### 2.1 Ts65Dn mice show genotype‐independent, sex‐specific differences in body and brain weight across their lifespan

Reports of trisomic Ts65Dn (herein referred to as 3N) mice body size have been inconsistent. For example, previous work suggests 3N mice typically exhibited reduced body size compared to their euploid (2N) littermates at 1–2 months of age (Bianchi et al., 2010; Fuchs et al., 2012) and at 15 months of age (Velazquez et al., 2013). However, it has also been documented that 3N and 2N males have similar body weight at 5 months (Fructuoso et al., 2018). More recently, an analysis of the updated strain of Ts65Dn also suggests that 2N vs. 3N body weights normalize by 1 month of age and morphometric changes might differ significantly between cohorts (Shaw et al., 2020). We therefore analyzed both body and brain weight in all mice prior to tissue harvesting. For body weight, we found a main effect of sex in 4‐month‐old mice (*F*_(1,8)_ = 17.071; *p* = 0.0033), with male mice being significantly heavier than female mice regardless of genotype. This sex‐specific difference in body weight was not observed at 7–8 months; however, we found a main effect of sex on brain weight at 7–8 months (*F*_(1,9)_ = 11.905; *p* = 0.0073) with females showing a higher brain weight than males, independent of genotype. Lastly, at 12 months of age, we observed a main effect of sex on body weight (*F*_(1,9)_ = 11.353; *p* = 0.0083) with males again weighing more than females independently of genotype. Collectively, these results suggest sex‐specific morphometric changes in body and brain weight as 3N mice age (Table 1) and emphasize the morphometric variability observed in Ts65Dn mice (Shaw et al., 2020).

**TABLE 1:** Genotype‐independent sex differences in body and brain weight across 4–12 months of age

Genotype	Body weight (g)	Brain weight (g)
4‐month‐old		
2N Male (*n* = 3)	35.157 ± 1.126	0.470 ± 0.012
3N Male (*n* = 3)	31.92 ± 0.497	0.460 ± 0.010
2N Female (*n* = 3)	25.307 ± 1.182	0.490 ± 0.012
3N Female (*n* = 3)	27.960 ± 2.874	0.490 ± 0.015
*Sex effect p‐value*	0.0033**	*0.1086*
7‐ to 8‐month‐old		
2N Male (*n* = 4)	45.602 ± 3.276	0.457 ± 0.016
3N Male (*n* = 3)	40.95 ± 1.499	0.457 ± 0.013
2N Female (*n* = 3)	46.307 ± 3.486	0.503 ± 0.009
3N Female (*n* = 3)	35.533 ± 5.196	0.500 ± 0.006
*Sex effect p‐value*	*0.5313*	0.0073**
12‐month‐old		
2N Male (*n* = 3)	44.927 ± 2.449	0.497 ± 0.009
3N Male (*n* = 3)	44.293 ± 1.225	0.487 ± 0.003
2N Female (*n* = 3)	35.783 ± 1.932	0.477 ± 0.003
3N Female (*n* = 4)	39.090 ± 2.294	0.477 ± 0.010
*Sex effect p‐value*	0.0083**	*0.1042*

Note

Only main effects of sex are shown as there were no significant genotype main effects and no interactions. 4‐month‐old female mice show a significantly lower body weight than male counterparts. 7‐8‐month‐old female mice show a significantly higher brain weight than male counterparts. 12‐month‐old female mice show a significantly lower body weight than male mice. Italicized are *p*‐values. Bold *p*‐value indicates statistically significant *p*‐values (**p < 0.01). Data reported as means ± SEM.

### 2.2 Soluble Aβ_40_ and Aβ_42_ are elevated in the frontal cortex and hippocampus of 3N mice by 4 months of age

**FIGURE 1:**
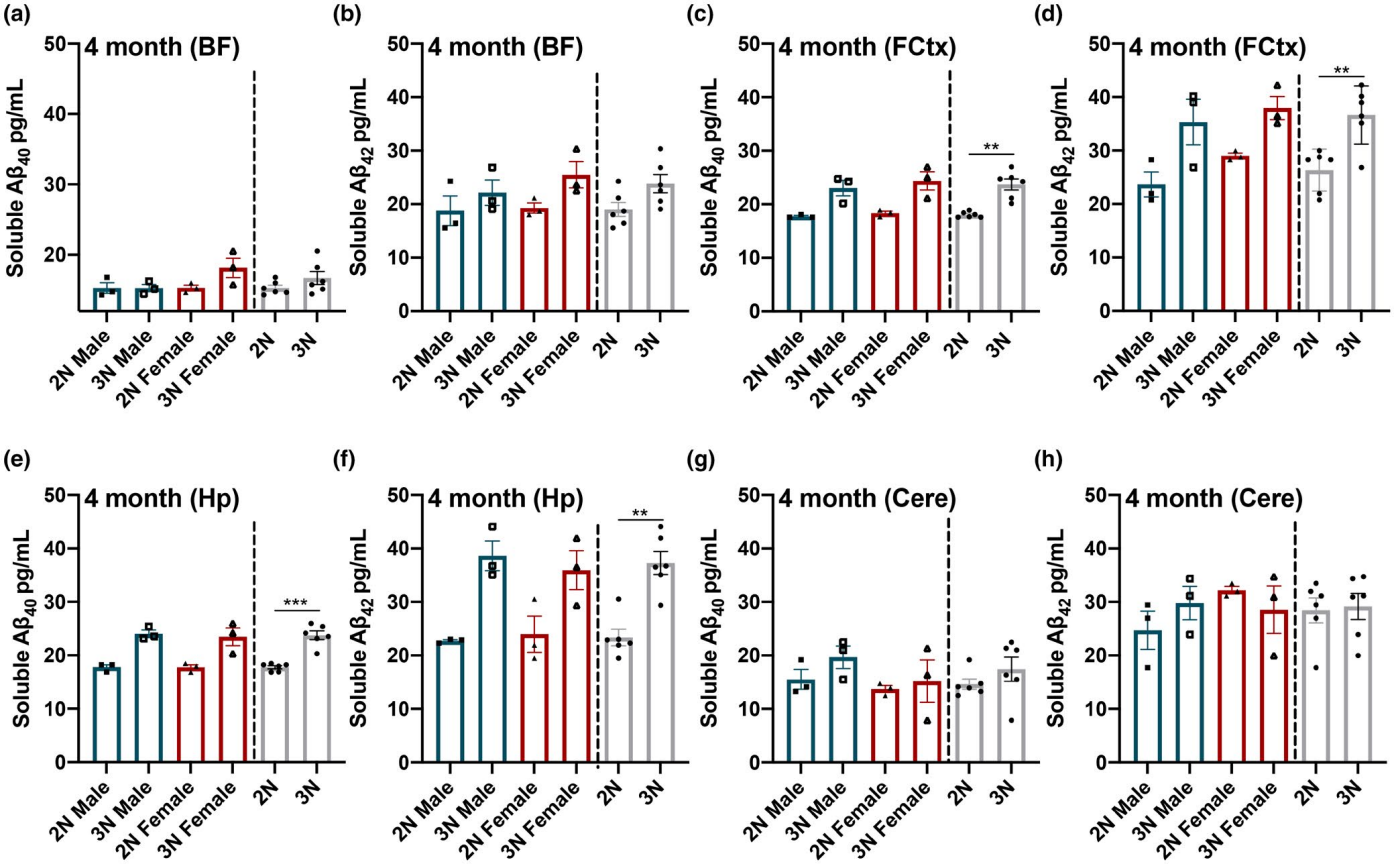
Soluble Aβ_40‐42_ levels at 4 months of age across 4 separate brain regions. (a, b) At 4 months of age, soluble Aβ_40‐42_ is not elevated in the BF of trisomic (3N) vs. diploid (2N) Ts65Dn mice. (c, d) 3N mice showed elevated Aβ_40‐42_ in both the FCtx and (e, f) the Hp compared to their age‐matched 2N controls. (g, h) Cere tissue showed no significant differences in Aβ_40‐42_ between 2N and 3N mice. Data are presented as means ± SEM. 2N vs 3N bars to the right illustrate the observed main effects of genotype. *p < 0.05, **p < 0.01, ***p < 0.001

Degeneration of basal forebrain cholinergic neurons (BFCNs) has been well documented in 3N mice by 4–6 months of age when compared with their 2N littermates (Cooper et al., 2001; Granholm et al., 2000). To determine if 3N mice show elevated soluble Aβ_40‐42_ coinciding with the onset of basal forebrain alterations, we measured the Aβ_40‐42_ present in soluble fractions from basal forebrain (BF), frontal cortex (FCtx), hippocampal (Hp), and cerebellar (Cere) homogenates (*n* = 6 3N and *n* = 6 2N; genotypes balanced by sex). We found no significant differences in BF levels of either Aβ_40_ or Aβ_42_ between 3N and 2N mice (Figure 1a,b). However, we saw a significant main effect of genotype for soluble Aβ_40_ (*F*_(1,8)_ = 24.518; *p* = 0.0011) and Aβ_42_ (*F*_(1,8)_ = 14.811; *p* = 0.0049) within the FCtx (Figure 1c,d) and significant main effect of genotype for soluble Aβ_40_ (*F*_(1,8)_ = 38.344; *p* = 0.0003) and Aβ_42_ (*F*_(1,8)_ = 24.062; *p* = 0.0012) within the Hp (Figure 1e,f), where 3N mice exhibited higher levels than the 2N counterparts. No sex effects were observed. Lastly, we found no significant differences in Cere levels of either Aβ_40_ or Aβ_42_ between 3N and 2N mice (Figure 1g,h). Thus, by four months of age 3N mice already exhibit measurable increases in soluble Aβ_40‐42_ species versus their 2N littermates, but only within the FCtx and Hp.

### 2.3 Sex differences in soluble Aβ_40_ and Aβ_42_ detected in 3N mice at 7–8 months of age

**FIGURE 2:**
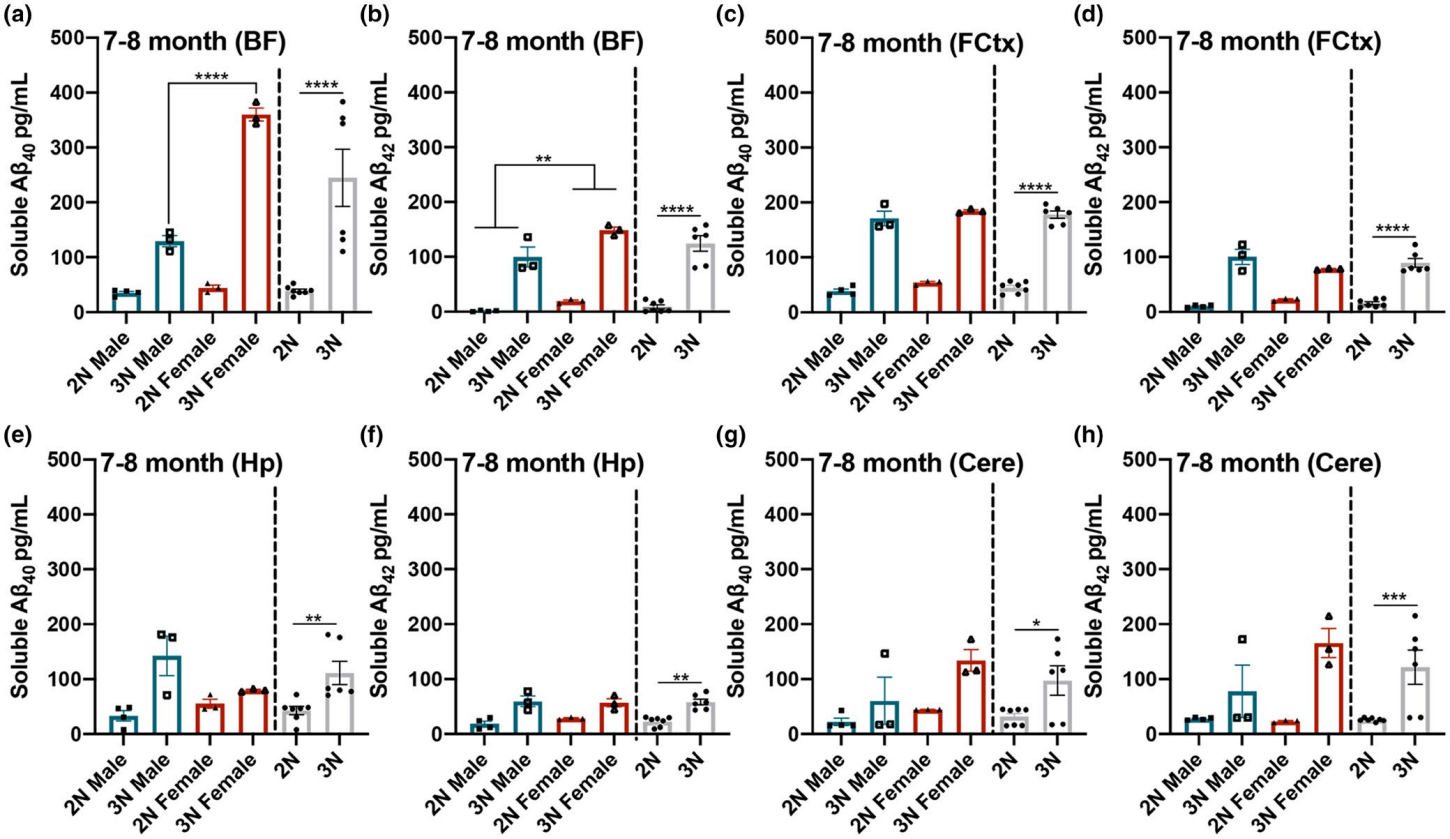
Soluble Aβ_40‐42_ levels are elevated in all brain regions of 3N mice compared to the 2N counterparts by 7–8 months of age, with observed sex‐specific differences. At 7–8 months of age, soluble Aβ_40‐42_ is elevated in 3N mice in all four brain regions. (a) In the BF, soluble Aβ_40_ is significantly elevated in 3N vs. 2N mice and in females vs. males; a significant interaction was also shown, with 3N females showing the highest levels. (b) Aβ_42_ is significantly elevated in 3N vs. 2N mice and in females vs. males. (c–h) 3N mice showed elevated Aβ_40‐42_ levels in the FCtx, the Hp, and the Cere compared to 2N mice. Data are presented as means ± SEM. 2N vs 3N bars to the right illustrate the observed main effects of genotype. **p < 0.01, ***p < 0.001, ****p < 0.0001

We next assessed levels of soluble Aβ_40_ and Aβ_42_ in 3N vs 2N mice in 7‐ to 8‐month‐old mice (*n* = 6 3N and *n* = 7 2N, balanced by sex) in the BF, FCtx, Hp, and Cere. Within the BF, we saw a significant main effect of genotype for soluble Aβ_40_ (*F*_(1,9)_ = 722.36; *p* < 0.0001) and Aβ_42_ (*F*_(1,9)_ = 173.999; *p* < 0.0001), where 3N mice exhibited elevated levels compared to 2N counterparts (Figure 2a,b). Additionally, we found a significant main effect of sex for soluble Aβ_40_ (*F*_(1,9)_ = 247.592; *p* < 0.0001) and Aβ_42_ (*F*_(1,9)_ = 14.988; *p* = 0.0038), where female mice showed higher levels of both fractions than male counterparts (Figure 2a,b). Moreover, there was a significant interaction between genotype and sex for soluble Aβ_40_ (*F*_(1,9)_ = 211.602; *p* < 0.0001); post hoc analysis revealed that 3N females had significantly higher Aβ_40_ than 3N males (*p* < 0.0001, Figure 2a).

In the FCtx, we observed a main effect of genotype for soluble Aβ_40_ (*F*_(1,9)_ = 381.150; *p* < 0.0001) and Aβ_42_ (*F*_(1,9)_ = 128.948; *p* < 0.0001), where 3N mice exhibited higher levels than 2N counterparts (Figure 2c,d). In the Hp, we observed a main effect of genotype for soluble Aβ_40_ (*F*_(1,9)_ = 13.986; *p* < 0.0046) and Aβ_42_ (*F*_(1,9)_ = 28.431; *p* = 0.0005), where 3N mice exhibited higher levels than 2N counterparts (Figure 2e,f). In the Cere, we observed a main effect of genotype for soluble Aβ_40_ (*F*_(1,9)_ = 8.461; *p* = 0.0173) and Aβ_42_ (*F*_(1,9)_ = 15.072; *p* = 0.0037), where 3N mice exhibited higher levels than 2N mice (Figure 2g,h). Collectively, these results indicate that, by 7–8 months of age, 3N mice exhibit widespread increased levels of soluble Aβ_40‐42_ species in all four brain regions; notably, female 3N mice show higher increases than 3N males in the BF, a region where sex differences in cholinergic neuron number have been detected (Kelley et al., 2014).

### 2.4 3N mice maintain significantly higher soluble Aβ_40_ and Aβ_42_ in all brain regions examined at 12 months

**FIGURE 3:**
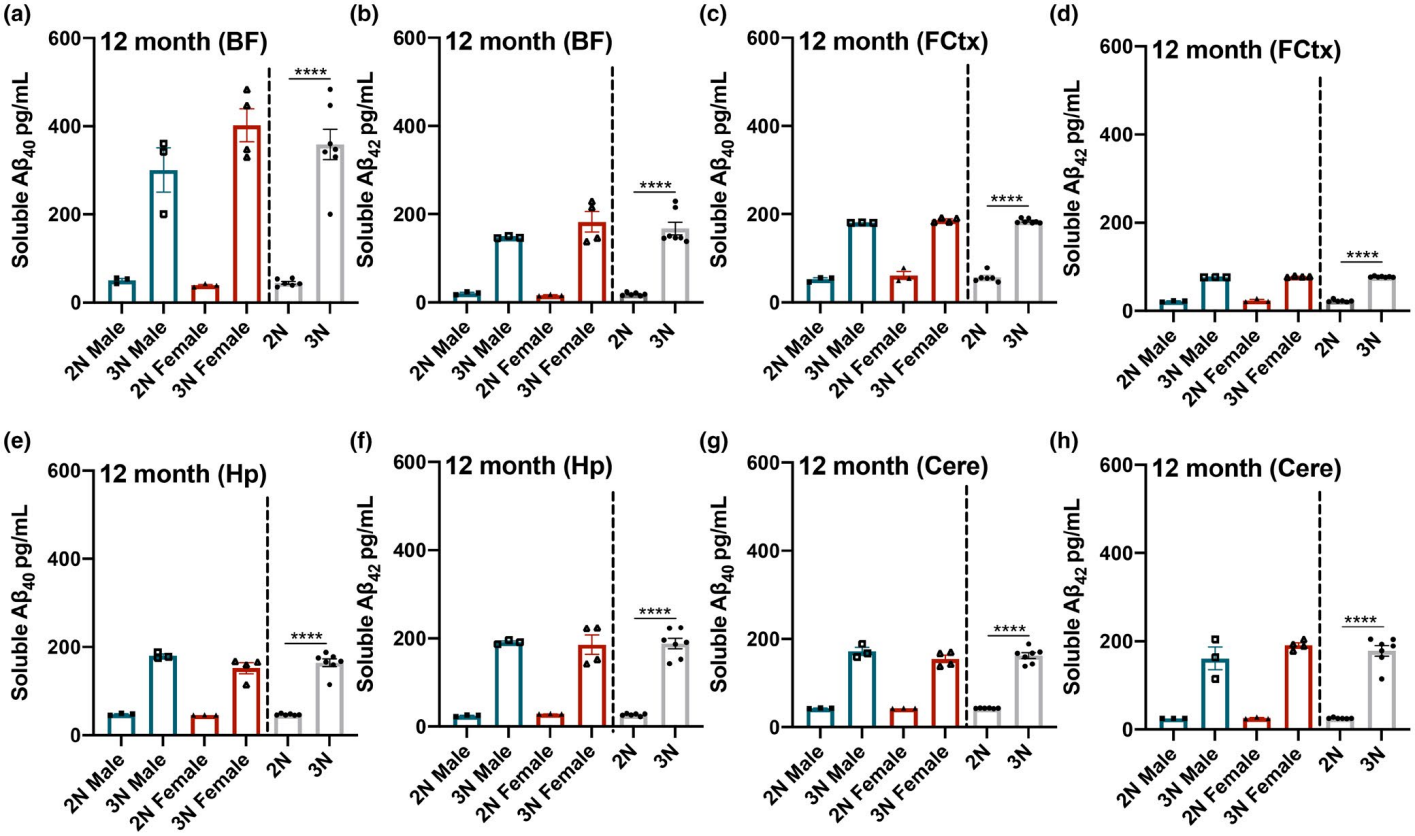
Soluble Aβ_40‐42_ levels are elevated in 3N mice compared to 2N counterparts at 12 months of age. At 12 months of age, soluble Aβ_40‐42_ is significantly elevated in 3N vs. 2N mice in all four brain regions: (a–h) BF, FCtx, Hp, and Cere. No significant sex effects or interactions were observed at this age. Data are presented as means ± SEM. 2N vs 3N bars to the right illustrate the observed main effects of genotype. ****p < 0.0001

While BFCN degeneration and cognitive deficits appear in Ts65Dn 3N mice by 4–6 months, these outputs show a sharp decline at 12 months (Powers et al., 2016). Thus, we next examined tissue homogenates at 12 months (*n* = 7 3N and *n* = 6 2N, balanced by sex). Within the BF, we found a significant main effect of genotype for soluble Aβ_40_ (*F*_(1,9)_ = 83.613; *p* < 0.0001) and Aβ_42_ (*F*_(1,9)_ = 94.153; *p* < 0.0001), where 3N mice show elevated levels compared to 2N (Figure 3a,b). Similarly, for the FCtx, we found a significant main effect of genotype for soluble Aβ_40_ (*F*_(1,9)_ = 767.405; *p* < 0.0001) and Aβ_42_ (*F*_(1,9)_ = 2,128.032; *p* < 0.0001), with elevations present in 3N mice (Figure 3c,d). Hp tissue likewise exhibited a significant main effect of genotype for soluble Aβ_40_ (*F*_(1,9)_ = 204.145; *p* < 0.0001) and Aβ_42_ (*F*_(1,9)_ = 128.132; *p* < 0.0001), with elevations present in 3N mice (Figure 3e,f). Lastly, Cere tissue showed a main effect of genotype for soluble Aβ_40_ (*F*_(1,9)_ = 325.441; *p* < 0.0001) and Aβ_42_ (*F*_(1,9)_ = 148.179; *p* < 0.0001), with elevations present in 3N mice (Figure 3g,h). No significant sex effects or interactions were observed by 12 months of age. Thus, while 3N mice continue to show higher levels of Aβ_40‐42_ by 12 months of age, any differences in soluble Aβ between sexes equalizes.

### 2.5 Soluble Aβ_40_ and Aβ_42_ levels show significant increases as a function of age in 3N mice

**FIGURE 4:**
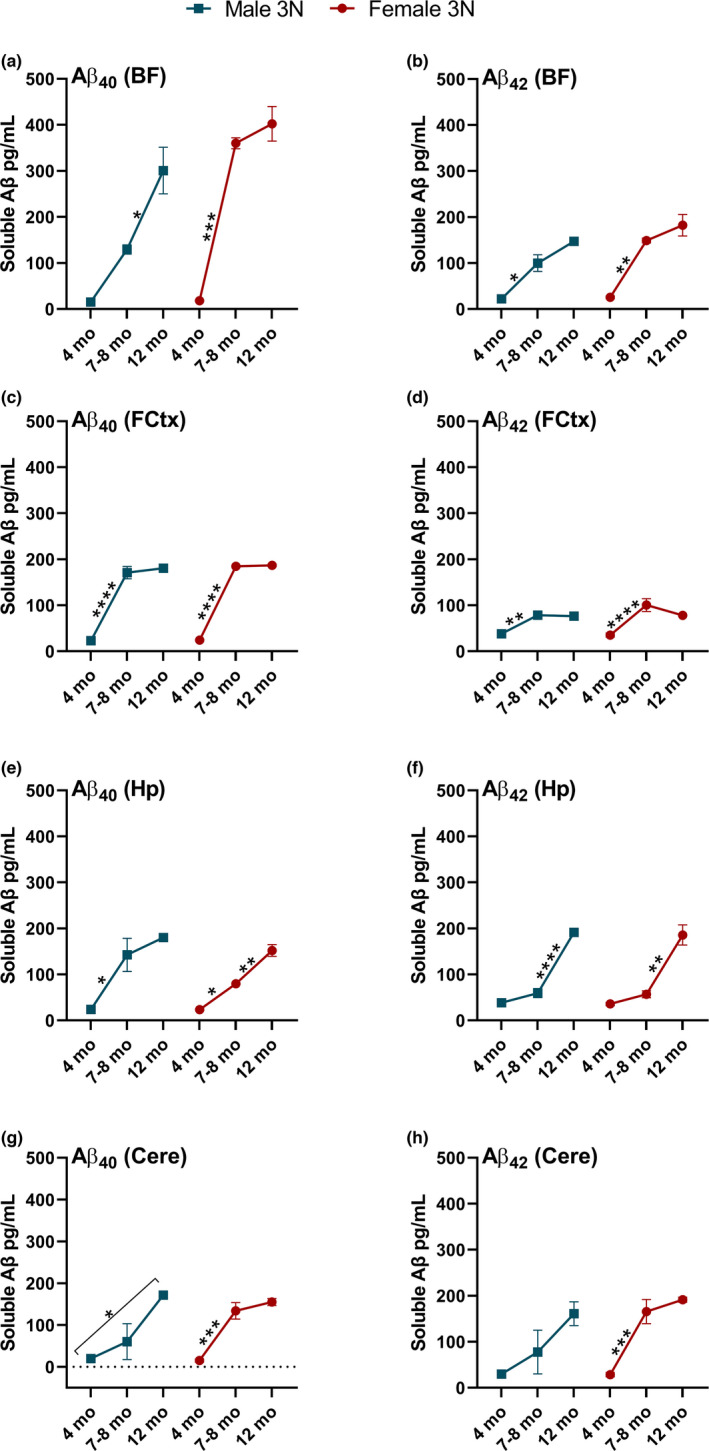
Soluble Aβ_40‐42_ in 3N mice changes as a function of age. (a) Aβ_40_ in the BF rises across age, with females showing a dramatic increase from 4 to 7–8 months. (b) Aβ_42_ in the BF rises significantly but less dramatically over time, particularly from 4 to 7–8 months. (c) Aβ_40_ rises significantly in the FCtx from 4 to 7–8 months before plateauing. (d) Aβ_42_ in the FCtx shows a significant increase between 4 and 7–8 months before plateauing. (e) Aβ_40_ in the Hp rises from 4 to 7–8 months in both sexes but also rises significantly in females from 7–8 to 12 months. (f) Aβ_42_ rises significantly in the Hp from 7–8 to 12 months. (g‐h) In the Cere, Aβ_40_ but not Aβ_42_ rises significantly across age in males, while significant increases in Aβ_40‐42_ are seen only in females from 4 to 7–8 months. Data are presented as means ± SEM. *p < 0.05, **p < 0.01, ***p < 0.001, ****p < 0.0001

Our data are the first characterization of differences in soluble Aβ_40‐42_ across multiple affected brain regions in 3N Ts65Dn mice versus their 2N controls. It is thus also helpful to determine how these potentially neurotoxic species change as the 3N mice age. In the BF, we found a significant main effect of age on soluble Aβ_40_ (*F*_(2,13)_ = 71.105; *p* < 0.0001) and Aβ_42_ (*F*_(2,13)_ = 49.398; *p* < 0.0001), a significant main effect of sex on soluble Aβ_40_ (*F*_(1,13)_ = 22.612; *p* = 0.0004) and Aβ_42_ (*F*_(1,13)_ = 5.973; *p* = 0.0295), and a significant age by sex interaction for Aβ_40_ alone (*F*_(2,13)_ = 7.564; *p* = 0.0066). Post hoc comparisons between time points revealed that the age‐specific and sex differences in Aβ_40_ (Figure 4a) were driven by a significant increase between 4 and 7–8 months for females (*p* = 0.0002) while males showed a significant increase later, between 7–8 and 12 months of age (*p* = 0.0198). Age‐specific elevations in Aβ_42_ were due to significant increases between 4 and 7–8 months in both males (*p* = 0.0059) and females (*p* = 0.0053). In the FCtx, we found a significant main effect of age on soluble Aβ_40_ (*F*_(2,13)_ = 558.519; *p* < 0.0001) and Aβ_42_ (*F*_(2,13)_ = 46.224; *p* < 0.0001) with no significant effects of sex or interaction effects. Post hoc comparisons revealed that the age‐specific effects were due to a significant increase between 4 and 7–8 months for Aβ_40_ (Figure 4c; both sexes: *p* < 0.0001) and Aβ_42_ (Figure 4d; males: *p* < 0.0001; females: *p* = 0017). In the Hp, we found a significant main effect of sex on soluble Aβ_40_ (*F*_(1,13)_ = 5.806; *p* = 0.0315) in addition to a significant main effect of age for both Aβ_40_ (*F*_(2,13)_ = 43.472; *p* < 0.0001) and Aβ_42_ (*F*_(2,13)_ = 82.84; *p* < 0.0001). Post hoc comparisons showed that, while the significant effect of age on Aβ_40_ levels (Figure 4e) was due to a significant increase between 4 and 7–8 months in males (*p* = 0.0209), female mice showed significant increases in Aβ_40_ between 4 and 7–8 months (*p* = 0.013) and between 7–8 to 12 months (*p* = 0.0023). The age‐specific elevations of Aβ_42_ in the Hp (Figure 4f) were instead driven by dramatic increases between 7–8 and 12 months for both males (*p* < 0.0001) and females (*p* = 0.0023). Lastly, in the Cere, we found a significant main effect of age on soluble Aβ_40_ (*F*_(2,13)_ = 29.955; *p* < 0.0001) and Aβ_42_ (*F*_(2,13)_ = 20.497; *p* < 0.0001). This effect of age on levels of Aβ_40_ (Figure 4g) in males, when tested by post hoc comparisons, was due to the overall difference between 4 and 12 months (*p* = 0.0163), while in females it was driven by the increase between 4 and 7–8 months (*p* = 0.0008). Similarly, post hoc comparisons revealed no specific age‐related increase in Aβ_42_ (Figure 4h) in males (*p* = 0.0753) while females showed a significant increase from 4 to 7–8 months (*p* = 0.0009). In conclusion, we show that when looking at 3N animals of different ages, soluble Aβ_40_ and Aβ_42_ significantly increase as a function of age, with most regions showing a sigmoidal pattern of accumulation, though they show temporal variation in each brain region and show sex‐specific effects particularly in the BF and Hp.

### 2.6 Aβ_40_ and Aβ_42_ levels in 3N mice are inversely correlated with APP levels in the BF and Hp, but not the FCtx or Cere

To determine whether the age‐ and sex‐specific differences in soluble Aβ_40‐42_ were related to the levels of full‐length APP, we quantified APP expression in the 3N mice via Western blot from the same homogenates used for our soluble Aβ_40‐42_ analysis. In the BF, we found a main effect of age on APP expression (*F*_(2,12)_ = 76.05; *p* < 0.0001; Figure S1A‐B) as APP levels decreased over time in both sexes. In the Hp, there was a significant main effect of age (*F*_(2,12)_ = 14.86; *p* = 0.0006), a significant main effect of sex (*F*_(1,12)_ = 11.31; *p* = 0.0056), and a significant age by sex interaction (*F*_(2,12)_ = 5.336; *p* = 0.0220; Figure S1C); post hoc analysis revealed that males had significantly lower APP levels than females at 7–8 months of age (*p* = 0.0093). There was no significant effect of age or sex on APP expression in the FCtx of 3N mice (Figure S1D). The Cere of 3N mice showed a significant main effect of age on APP expression (*F*_(2,12)_ = 6.170; *p* = 0.0144) and a significant age by sex interaction (*F*_(2,12)_ = 4.033 *p* = 0.0457; Figure S1E); post hoc analysis revealed that males at 7–8 months differed significantly from females at 4 months (*p* = 0.0157). Overall, these results show that APP levels in 3N mice of the updated Ts65Dn 5252 strain decrease as a function of time in the BF and Hp, but fluctuate with more complexity in the FCtx and Cere.

**FIGURE 5:**
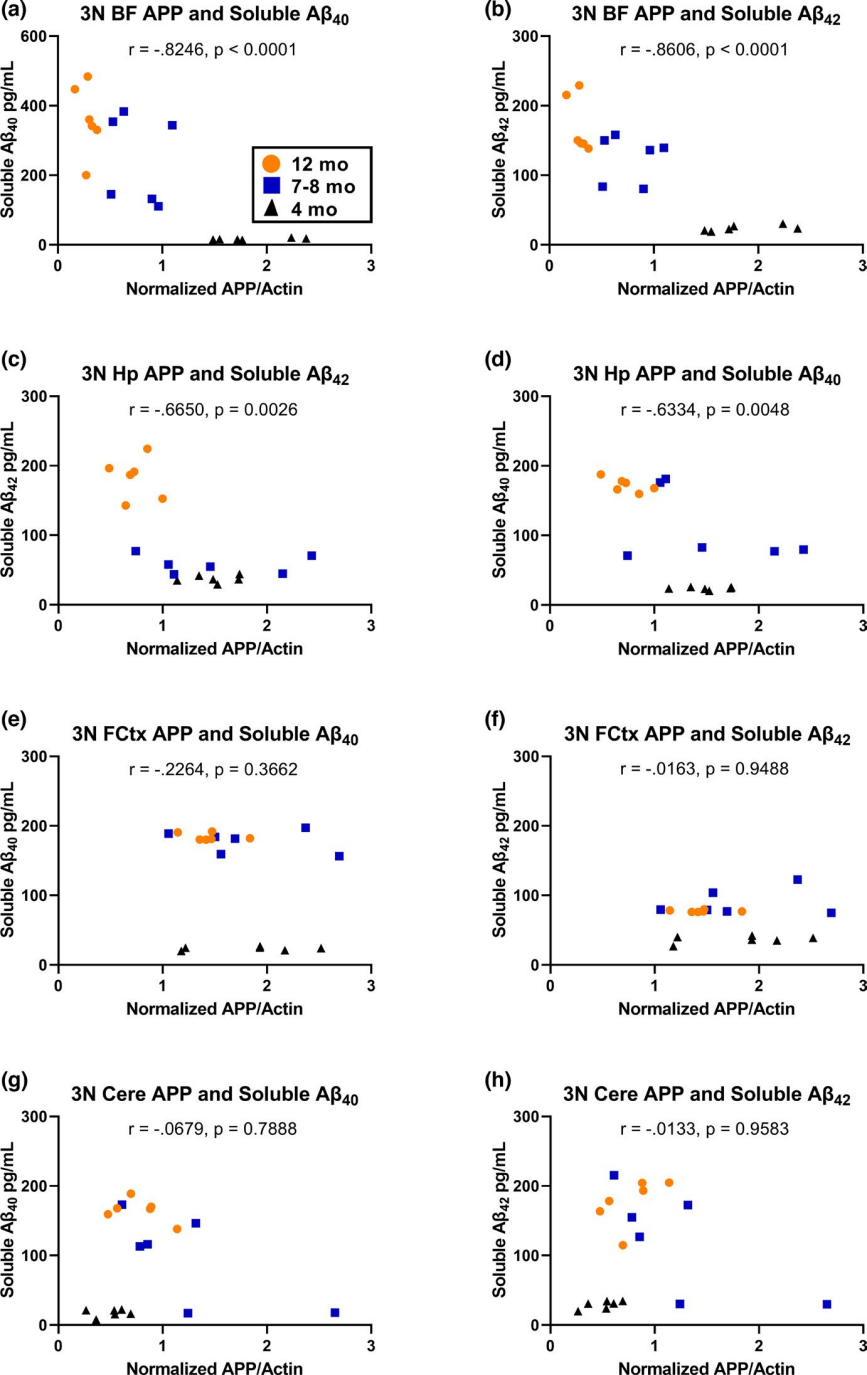
Full‐length APP is inversely correlated with soluble Aβ_40‐42_ in the BF and Hp. (a, b) Soluble Aβ_40_ and Aβ_42_ are each significantly inversely correlated with APP levels in the BF of 3N mice. (c, d) Soluble Aβ_40_ and Aβ_42_ are each significantly inversely correlated with APP levels in the Hp of 3N mice. (e–h) Neither soluble Aβ_40_ nor Aβ_42_ in the FCtx or Cere are correlated to APP levels in 3N mice

To determine how the levels of APP are related to the observed increases in soluble Aβ_40_ and Aβ_42_ in 3N mice as a function of age, we next correlated the level of APP at each time point and brain region to the changes in soluble Aβ_40_ and Aβ_42_ in each animal. In the BF, both Aβ_40_ (*r* = −0.8246; *p* < 0.0001) and Aβ_42_ (*r* = −0.8606; *p* < 0.0001) showed significant inverse correlations with APP levels (Figure 5a,b). Similarly, within the Hp both Aβ_40_ (*r* = −0.6334; *p* = 0.0048) and Aβ_42_ (*r* = −0.6650; *p* = 0.0026) showed significant inverse correlations with APP levels (Figure 5c,d). Within the FCtx, there was no significant relationship between Aβ_40_ and APP (*r* = −0.2264; *p* = 0.3662) or Aβ_42_ and APP (*r* = −0.01629; *p* = 0.9488; Figure 5e,f). Likewise, the levels of APP in the Cere tissue were not significantly correlated with Aβ_40_ (*r* = −0.06793; *p* = 0.7888) or Aβ_42_ (*r* = −0.01328; *p* = 0.9583; Figure 5g,h). Thus, in 3N Ts65Dn mice, APP levels decrease as soluble Aβ_40‐42_ levels increase, but only in the BF and Hp.

## 3 DISCUSSION

Our results show that soluble Aβ_40‐42_ production in 3N Ts65Dn mice changes in an age‐dependent manner across four brain regions that hold relevance in the context of DS and AD—the basal forebrain, frontal cortex, hippocampus, and cerebellum. We observed significantly increased soluble Aβ_40_ and Aβ_42_ within the frontal cortex and hippocampus of 3N mice by 4 months of age when compared to their 2N littermates, and significant increases in 3N vs. 2N mice in all examined brain regions by 7–8 months and lasting as long as 12 months of age. While soluble Aβ_40_ and Aβ_42_ do not advance in this mouse model into extracellular Aβ plaques, soluble Aβ species, with Aβ_42_ in particular, are capable of self‐assembly into oligomers that may directly drive neuronal dysfunction (reviewed in Li & Selkoe, 2020). The elevation of soluble Aβ_40‐42_ in the frontal cortex and hippocampus of 3N mice by only 4 months of age occurs in a similar timeframe to previously observed impairments in prefrontal–hippocampal circuit function and performance on memory tasks (Alemany‐González et al., 2020), which may suggest a relationship between the two. Furthermore, it is notable that maintenance of the basal forebrain cholinergic neurons is reliant on the internalization and retrograde transport of nerve growth factor (NGF) protein from their terminal fields in both the frontal cortex and hippocampus, and NGF dysregulation has been implicated in DS (Hampel et al., 2018). To this end, in 3N mice, impaired NGF transport from the hippocampus back to the basal forebrain has been demonstrated at 6 months of age (Cooper et al., 2001). Additionally, a report has shown that soluble Aβ acts as an antagonist for NGF, thereby contributing to dysregulation (Arevalo et al., 2009). Our data suggest that elevated soluble Aβ_40‐42_, evident in the hippocampus and frontal cortex at 4 months precedes the onset of NGF transport dysfunction in cholinergic neurons that then extends to dysfunction within the basal forebrain itself, suggesting a potential link between the two.

The increased Aβ_40‐42_ we observed in the basal forebrain by 7–8 months and continued elevation in the hippocampus coincides with the onset of spatial learning and memory deficits mediated by the septo‐hippocampal circuit in 3N mice (Granholm et al., 2000) and attentional dysfunction mediated by basal forebrain‐frontal cortex circuitry (Powers et al., 2016). Interestingly, we also observed a sex difference in the accumulation of Aβ_40_ and Aβ_42_ in the basal forebrain of 3N animals between 4 and 7–8 months of age, with females’ levels increasing dramatically from 4 to 7–8 months. Previous work has shown sex differences in cholinergic basal forebrain neuron number of 3N mice by 5–8 months of age, where females show a reduced neuron number compared to age‐matched males (Kelley et al., 2014). Thus, the increase in soluble Aβ_40_ and Aβ_42_ within the basal forebrain of females at this age coincides with the sex‐specific reduction in cholinergic neurons, suggesting a potential contributing role for soluble Aβ species more directly in the deterioration of neurons in this region in Ts65Dn mice. Human females show a higher incidence and earlier onset of AD, with some work suggesting their basal forebrain cholinergic neurons may be more sensitive to degeneration, possibly due to decreased NGF receptor expression when compared to males (Counts et al., 2011). However, the nature of sex differences in AD within human DS is inconclusive (Martini et al., 2021). Further work is thus warranted to determine how sex and soluble Aβ elevation interact in the context of neurodegeneration in the basal forebrain, both in the Ts65Dn model and in human AD and DS.

All brain regions investigated here exhibited high levels of soluble Aβ_40‐42_ at 12 months of age, with two notable observations: (1) The basal forebrain showed the highest overall accumulation of Aβ_40_ and Aβ_42_ by 12 months in all the brain regions studied, and (2) In the Hp, soluble Aβ_42_ increased more dramatically in the interval between 7–8 and 12 months than in the interval between 4 and 7–8 months. Meta‐analysis of water maze performance, which assesses learning and memory, showed that the performance of 3N mice takes a steep decline during the 7–8 to 12‐month interval (Hamlett et al., 2016), while attentional dysfunction worsens in this mouse model around 12 months (Powers et al., 2016). Given the role of the basal forebrain in mediating learning and memory functions of the hippocampus and in attentional behavior of the frontal cortex, further investigation is warranted to determine the role elevated basal forebrain Aβ_40‐42_ levels play in the context of these cognitive deficits. Similarly, the steep climb in hippocampal levels of the more neurotoxic Aβ_42_ may be an important consideration when investigating hippocampal function as 3N mice age. While the data presented here are not longitudinal, it is also interesting to note that trajectories of Aβ_40‐42_ elevation follow a sigmoidal pattern, where the BF and Cere are just beginning to plateau, the FCtx has perhaps fully reached its plateau, and the hippocampus is in a rising phase without evidence of leveling off. While these data demonstrate region‐specific temporal latency, the apparent sigmoidal pattern is of note in the context of previous neuroimaging work suggesting that the timing of Aβ deposition also follows a sigmoidal pattern of accumulation in AD despite differing between brain regions in the timing of onset (Jack et al., 2013).

The inverse correlation between APP levels and soluble Aβ_40‐42_ observed here in the basal forebrain and hippocampus suggests that, as 3N animals age, more of the available full‐length APP is converted to soluble Aβ_40_ and Aβ_42_ in these regions specifically. This significant finding may lie in the balance between the activity of α‐, β‐, and γ‐secretases and/or the impaired export and subsequent clearance of extracellular soluble Aβ_40‐42_ by microglia (Yuyama et al., 2012). Further work is needed to explore the variations in APP processing as a function of age and brain region in 3N mice, as well as the interaction between increased soluble Aβ_40‐42_ and other drivers of neuronal dysfunction, particularly in the basal forebrain. Furthermore, it may be worthwhile to explore changes in soluble Aβ_40‐42_ over time in additional AD‐relevant brain regions such as the striatum. Collectively, this work characterizes soluble Aβ_40‐42_ alterations in a temporal and spatial manner in both male and female Ts65Dn mice, thereby informing scientists about the sex‐based differences when assessing amyloid pathologies in this model.

## 4 EXPERIMENTAL PROCEDURES

### 4.1 Animals

The B6EiC3Sn.BLiA‐Ts(1716)65Dn/DnJ (Ts65Dn) mice (Jackson Laboratory Strain #005252) used in this study have been described previously (Ahmed et al., 2012; Costa et al., 2010). 3N founder dams were purchased in 2019 from Jackson Laboratory. Subsequently, the colony has been maintained by breeding 3N female Ts65Dn mice to males on the B6EiC3Sn.BLiAF1/J background (Jackson Laboratory Strain #003647) purchased as needed for colony breeding cohorts of 5–9 dams at a time. 3N mice are segmentally trisomic, containing a translocation chromosome with segments of mouse Chromosomes 16 and 17, containing approximately 2/3 of HSA21 gene orthologues (Reinholdt et al., 2011) corresponding to the DSCR (Antonarakis et al., 2004); their 2N littermates are used as controls. All protocols were approved by the Institutional Animal Care and Use Committee of Arizona State University and conform to the National Institutes of Health Guide for the Care and Use of Laboratory Animals. 2N and 3N littermates were same‐sex group housed (4–5 mice per cage) after weaning at 21 days of age. To confirm genotype, tail snips were collected and digested in STE Buffer with proteinase K, followed by DNA precipitation in isopropanol; genotypes were verified using the following PCR primers:

- Mut Forward: GTGGCAAGAGACTCAAATTCAAC
- Mut Reverse: TGGCTTATTATTATCAGGGCATTT
- Int Forward: CTAGGCCACAGAATTGAAAGATCT
- Int Reverse: GTAGGTGGAAATTCTAGCATCATCC

**FIGURE 6:**
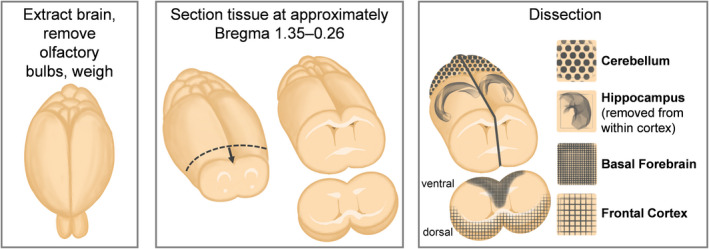
Brain region dissection protocol. Schematic of tissue extraction protocol showing isolation of the basal forebrain (BF), frontal cortex (FCtx), hippocampus (Hp), and cerebellum (Cere) from whole mouse brain

A total of 38 mice balanced by sex and genotype were euthanized at three time points—4 months (*n* = 12), 7–8 months (*n* = 13), and 12 months (*n* = 13) of age. Mice were weighed, anesthetized via intraperitoneal injection of ketamine (120 mg/kg body weight) and xylazine (6 mg/kg), and then transcardially perfused with phosphate‐buffered saline. After recording post‐mortem brain weight, we dissected the basal forebrain in the medial septal/ventral diagonal band region as described previously (Alldred et al., 2021) by taking an approximately 1.5 mm thick coronal section at approximately Bregma 1.35–0.26, between the rostral‐most appearance of the anterior forceps of the corpus callosum and where the anterior commissure crosses midline (Figure 6). Frontal cortex samples were collected bilaterally from directly above the basal forebrain; hippocampal, cortical, and cerebellar tissue were isolated bilaterally from the remainder of the brain. All tissues were flash‐frozen on dry ice and stored at −80°C until use.

### 4.2 Protein extraction, ELISA, immunoblotting

Basal forebrain, frontal cortex, hippocampus, and cerebellum were homogenized in a T‐PER tissue protein extraction reagent supplemented with protease (Roche Applied Science, IN, USA) and phosphatase inhibitors (Millipore, MA, USA). The homogenized tissues were centrifuged at 4 °C for 30 min, and the supernatant (soluble fraction) was stored at −80 °C. Soluble fractions of mouse Aβ_40_ and Aβ_42_ in the homogenates were detected using the commercially available ELISA kits (Invitrogen‐Thermo Fisher Scientific KMB3481 and KMB3441, respectively) as previously described (Winslow et al., 2021). All samples were run in duplicate wells. For immunoblotting, 30 μg of total protein from the regional homogenates was separated under reducing conditions and blotted as previously detailed (Winslow et al., 2021). The following Invitrogen antibodies were purchased and used: APP (Dilution 1:500, Catalog #14‐9749–82), β‐Actin (Dilution 1:5,000, Catalog #PA116889). Quantitative analyses of the Western blots were obtained by normalizing the intensity of the protein of interest with its own loading control β‐actin, within each blot. For quantitative comparisons between blots, a control sample was loaded in each blot, and quantitative values were averaged across all blots. Licor Image Studio software was used to quantify the intensity of the bands of interest. The experimenter was blinded to the group allocations.

### 4.3 Statistical analyses

Data from 2N vs 3N animals at each time point, as well as for 3N animals only looking across all time points, were analyzed using two‐way ANOVA with Bonferroni corrections for multiple comparisons using StatView 5.0.1 (SAS Institute) and GraphPad Prism (v9.2.0) with significance set at *p* < 0.05. Descriptive statistics were computed for each time point and no violations of assumptions required alternative testing procedures. Correlations between APP protein and soluble levels of Aβ_40‐42_ for each brain region were analyzed using Pearson's r in GraphPad Prism (v9.2.0) with significance set at *p* < 0.05.

## CONFLICT OF INTEREST

The authors declare that the research was conducted in the absence of any commercial or financial relationships that could be construed as a potential conflict of interest.

## AUTHOR CONTRIBUTIONS

Savannah Tallino, MS: involved in experimental design, animal harvesting and tissue preparation, statistical analysis, figure creation, and writing and editing the manuscript. Wendy Winslow, BS: involved in ELISA, and writing and editing the manuscript. Samantha K Bartholomew, BS: involved in tissue preparation, Western blotting and analysis, and editing the manuscript. Ramon Velazquez, PhD: involved in experimental design, funding, statistical analysis, figure creation, and writing and editing the manuscript.

## Supporting information

Fig S1

## Data Availability

The raw data supporting the conclusions of this article will be made available by the authors, without undue reservation.
