# Supplementary figures and images for: Temporal and brain region‐specific elevations of soluble Amyloid‐β40‐42 in the Ts65Dn mouse model of Down syndrome and Alzheimer’s disease
Source: Aging Cell. 2022 Mar 15;21(4):e13590. doi: 10.1111/acel.13590 (PMC9009111; doi:10.1111/acel.13590)

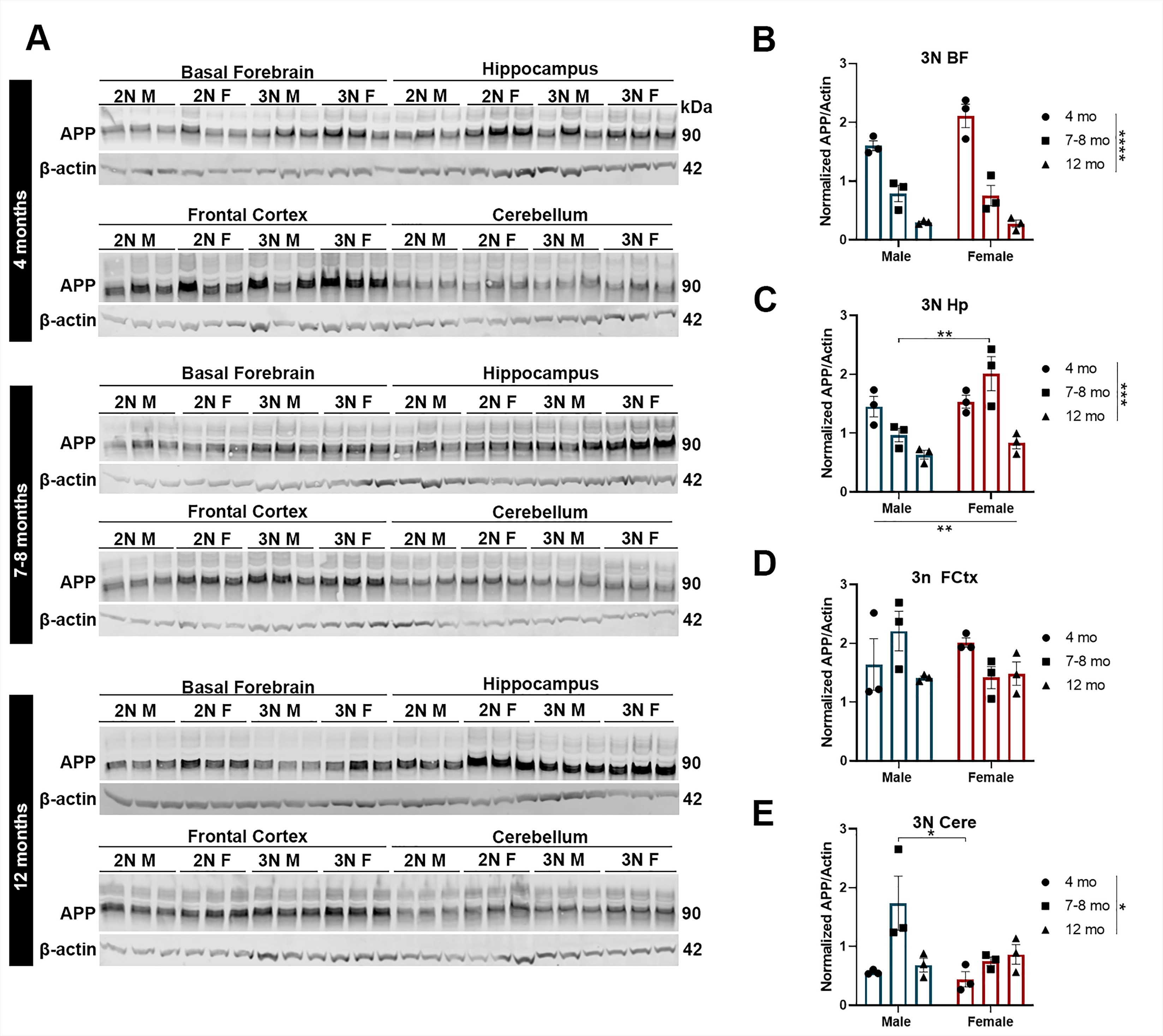

Supplement: Supplementary file 1 — Fig S1 [file ACEL-21-e13590-s001.tif]
